# Analysis of Clinical Factors Associated with Retinal Morphological Changes in Patients with Primary Sjögren's Syndrome

**DOI:** 10.1371/journal.pone.0157995

**Published:** 2016-06-21

**Authors:** Jee Myung Yang, Mi Sun Sung, Yong Sok Ji, Hwan Heo, Sang Woo Park

**Affiliations:** 1 Department of Ophthalmology, Chonnam National University Medical School and Hospital, Gwangju, Republic of Korea; 2 Graduate School of Medical Science and Engineering, Korea Advanced Institute of Science and Technology (KAIST), Daejeon, Republic of Korea; 3 Center for Creative Biomedical Scientists at Chonnam National University, Gwangju, Republic of Korea; National Institute of Dental and Craniofacial Research, UNITED STATES

## Abstract

**Purpose:**

To investigate clinical factors associated with abnormal retinal morphologies in patients with primary Sjögren's syndrome (pSS).

**Methods:**

One-hundred-thirty patients with pSS who underwent immunoserological tests, minor salivary gland biopsies, and optical coherence tomography examinations were retrospectively analyzed. Risk factors for abnormally reduced peripapillary retinal nerve fiber layer (pRNFL) and macular ganglion cell–inner plexiform layer (mGCIPL) thicknesses were evaluated, as well as the correlation between clinical factors and pRNFL and mGCIPL thicknesses.

**Results:**

Anti-Sjögren's syndrome type B (SSB) antibody positivity (*P* = 0.048) was identified as a risk factor associated with abnormally reduced pRNFL thickness, and anti-SSB positivity (*P* = 0.005) and erythrocyte sedimentation rate (ESR) level (*P* = 0.031) were identified as risk factors associated with an abnormally reduced mGCIPL thickness as revealed by multivariate logistic regression analysis. There was a significant negative correlation between anti-SSB antibody levels and the thickness of pRNFL and mGCIPL. The thicknesses of pRNFL and mGCIPL were significantly reduced in anti-SSB–positive eyes when compared to anti-SSB–negative eyes (*P* < 0.05). However, histopathologic grading was not associated with the pRNFL and mGCIPL thicknesses.

**Conclusion:**

Anti-SSB antibody positivity and ESR levels may be useful for predicting an abnormally reduced pRNFL or mGCIPL thickness in patients with pSS. Our results may provide clinical evidence to substantiate the association between aberrant autoimmunity and inner retinal changes in patients with pSS.

## Introduction

Sjögren’s syndrome (SS) is a chronic autoimmune disorder characterized by broad organ-specific and systemic manifestations, including sicca symptoms and a wide range of extraglandular manifestations such as arthritis, vasculitis, peripheral neuropathy, and central nervous system involvement [1]. The extraglandular manifestations are closely related to the patient’s aberrant immune status and disease activity [1,2]. The presence of antibodies (anti-Sjögren's syndrome type A (SSA)/Sjögren's syndrome type B (SSB)) and salivary gland inflammation assessed by the lymphocytic focus score (LFS) are both included in the classification criteria and diagnosis of primary Sjögren’s syndrome (pSS) [3]. Higher titers of anti-SSA and anti-SSB antibodies in serum are associated with an earlier disease onset and more frequent extraglandular complications [4–6]. Similarly, intense salivary gland inflammation has been related to the presence of extraglandular systemic manifestations in pSS, indicating that such patients comprise a distinct subgroup with more severe disease and aberrant immune responses [7–9].

In terms of retinal neurodegeneration, the role of the immune system in the loss of retinal ganglion cells (RGC) has long been a subject of controversy. Accumulating evidence suggests that a failure in the regulation of immunity or aberrant autoimmunity can initiate or aggravate the loss of RGC [10–13]. Our recent study has revealed that increased positivity for autoantibodies is associated with thinning of the peripapillary retinal nerve fiber layer (pRNFL) or macular ganglion cell–inner plexiform layer (mGCIPL) in patients with pSS [14]. However, our previous study did not quantify serum autoantibodies and did not evaluate other clinical factors, including histopathologic severity. Moreover, it excluded patients with an abnormally reduced pRNFL or mGCIPL thickness, which was measured by spectral domain optical coherence tomography (SD-OCT).

Evaluating inner retina in patients with pSS may be an indispensable part of clinical examinations because pSS patients are prone to develop RGC damage during the treatment course; after the diagnosis of pSS, most of the physicians use long-term retinal toxic medications such as hydroxychloroquine (HCQ), which is known to have irreversible and potentially blinding retinal damage, that occurs initially in RGCs [15–18]. Therefore, the aim of this study was to investigate changes in the morphology of retinas by measuring the thicknesses of pRNFL and mGCIPL in patients with pSS using SD-OCT and to assess the relationship between these morphological changes of the retina and various clinical factors, including histopathological and serological features that are routinely examined in clinical practice.

## Materials and Methods

### Subjects

This study enrolled 130 patients with pSS recruited from the Department of Ophthalmology and Rheumatology, Chonnam National University Medical School and Hospital between February 2012 and September 2014. Seventy-three age and sex matched normal controls were recruited from the healthy population. The study was conducted in accordance with the Declaration of Helsinki. Written informed consent was obtained from all subjects, and the protocol was approved by the Institutional Review Board of Chonnam National University Hospital.

All patients underwent detailed ophthalmologic examinations, which included: (1) medical and family histories, (2) testing for visual acuity, manifest refraction, intraocular pressure (IOP) measurements using Goldmann applanation tonometry (GAT), (3) slit lamp biomicroscopy with gonioscopy, (4) detailed optic nerve head (ONH) and RNFL examination with disc photography and red-free RNFL photography (TRC-NW6S camera; Topcon Corporation), and (5) visual field (VF) examination with a Humphrey field analyzer (Carl Zeiss Meditec Inc., Dublin, CA) and Swedish interactive threshold algorithm (SITA) 30–2 test. ONH and RNFL examinations were performed by a glaucoma specialist (S.W.P.). For data analysis, refractive error was converted to spherical equivalent (SE) refractive error.

Inclusion criteria were as follows: (1) confirmed diagnosis of pSS, (2) ≥18 years of age, (3) IOP of <21 mm Hg on diurnal testing with GAT, (4) best-corrected visual acuity (BCVA) ≥20/40, (5) SE refractive error between −6.0 and +4.0 diopters, cylinder correction within ±3.0 diopters, and (6) open anterior chamber angle on slit-lamp examination by gonioscopy. pSS diagnosis was based on the 2002 revised American-European Consensus Group (AECG) classification, which requires at least four out of six criteria, or three out of four objective criteria, to be met [3]. The six criteria include the following: (1) subjective and objective ocular dryness, (2) subjective and objective oral dryness, (3) presence of anti-SSA or anti-SSB, and (4) positive minor salivary gland biopsy. Patients who present features of pSS and other autoimmune connective tissue diseases (e.g rheumatoid arthritis) were considered to be secondary SS and excluded from this study.

Exclusion criteria included a history of any retinal or optic nerve disease, ocular trauma, ocular surgery, and systemic medical conditions other than pSS. Patients were excluded if they presented with glaucomatous optic nerve configuration, defined as one of the following: (1) vertical cup-to-disc ratio (CDR) ≥ 0.7, (2) asymmetry between the vertical CDR of both eyes > 0.2, (3) the presence of focal neural rim notching, (4) generalized loss of the neural rim, or (5) visible RNFL defect on red-free fundus photography with corresponding VF defects. Patients presenting with the disease in the central nervous system (CNS) were excluded based on the results of the neurologic examination and neuroimaging such as CT and MRI performed at the department of neurology to avoid the effect of trans-neuronal retrograde degeneration which might bias the results of our study [19,20]. In addition, to rule out potential retinal damage inflicted by systemic medication, patients taking a cytotoxic drug upon examination (eg, hydroxychloroquine or chloroquine) were excluded. When both eyes satisfied the inclusion criteria, the right eye of each subject was chosen for data analysis to avoid bias caused by an association between the eyes of each subjects [21,22].

### Optical Coherence Tomography Measurements

All OCT measurements were performed by one experienced examiner who was masked to other clinical information. After pupil dilation with tropicamide 1% and phenylephrine 2.5%, patients were asked to keep their eyes fixed on an internal target and both eyes of each patient were scanned with Cirrus HD-OCT (Carl Zeiss Meditec Inc.). Three scans per eye were obtained on the same day. If the quality of the initial scan was insufficient, patients’ eyes were lubricated with artificial tears prior to the examination. Only scans with signal strength ≥7 and without eye movements or blinking artifacts were used for data analysis. Any scan showing an overt decentration of the measurement circle or apparent segmentation error (eg, wrong identification of the retinal or retinal pigment epithelium plane), was excluded.

**Fig 1:**
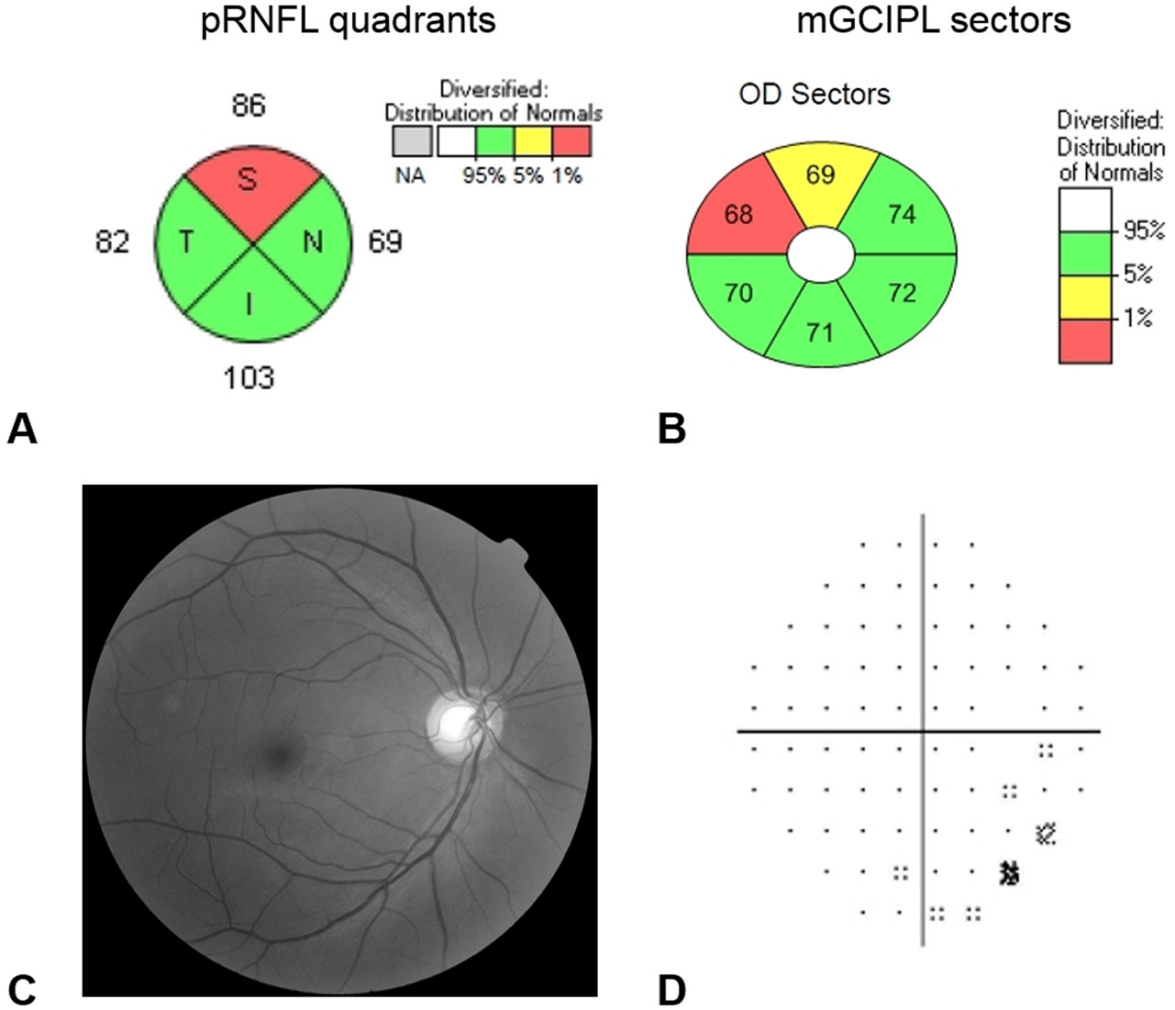
Representative case of anti-SSB positive patient (right eye of 51 year-old female) who showed abnormally reduced thickness of the peripapillary retinal nerve fiber layer (pRNFL) and macular ganglion cell–inner plexiform layer (mGCIPL). Color coding is as follows: green = normal range, yellow = below the 5th percentile of normal distribution and red = below the 1st percentile of normal distribution. (A) pRNFL thickness below the 5th percentile in average thickness and in at least one segment of the quadrant was considered as an indicator of abnormally reduced pRNFL thickness. (B) mGCIPL thickness below the 5th percentile in average and minimum thickness and in at least one of six sectors was considered as an indicator of abnormally reduced mGCIPL thickness. The patient showed nonspecific findings in RNFL photo (C) and visual field exam (D).

Both a macular scan (Macular Cube 200 × 200 protocol) and an optic disc scan (Optic Disc Cube 200 × 200 protocol) were performed on each patient. The ganglion cell analysis (GCA) algorithm in the 6.0 software version of Cirrus HD-OCT was used to analyze the average, minimum (the lowest mGCIPL thickness over a single meridian crossing the annulus), and six sectoral (superotemporal, superior, superonasal, inferonasal, inferior, and inferotemporal) thicknesses of the mGCIPL. Quadrant pRNFL thickness values in the optic disc scan were also analyzed. The macular cube and optic disc cube protocols analyze the pRNFL and mGCIPL thickness values and compare them with the internal age-matched normative database. The values are color-coded as follows: green = normal range, yellow = below the 5th percentile of normal distribution, and red = below the 1st percentile of normal distribution. For data analysis, an abnormally reduced thickness was defined as the thickness values below the 5th percentile (i.e., both yellow and red codes) [23]. pRNFL thickness below the 5th percentile in average thickness and in at least one segment of the quadrant was considered as abnormally reduced [24]. Similarly, mGCIPL thickness below the 5th percentile in average and minimum thickness, and in at least one of the six sectors was considered as abnormally reduced (Fig 1).

### Serologic Testing

Serologic testing was performed at a single clinical laboratory (Chonnam National University Hospital Laboratory) and included: (1) complete blood count, (2) screening for antinuclear antibody (ANA), and (3) tests for anti-SSA and anti-SSB antibodies, erythrocyte sedimentation rate (ESR), C-reactive protein, immunoglobulin (Ig) G, Ig A, Ig M, and rheumatoid factor. Enzyme-linked immunosorbent assay (ELISA) kits (Alegria, Orgentec Diagnostika, Mainz, Germany) were used for qualitative and quantitative measurements of the autoantibodies to extractable nuclear antigens anti-SSA (60-kDa and 52-kDa) and anti-SSB (48-kDa). Screening for ANA was performed by an indirect immunofluorescence test.

### Histopathologic Examination

All patients underwent minor salivary gland biopsies in the lower lip performed by an experienced otolaryngologist. The biopsy specimens were formalin-fixed, paraffin-embedded, and stained with hematoxylin and eosin. LFS was recorded as the number of lymphocytic foci (defined as aggregates of ≥50 mononuclear cells) per 4 mm^2^ of salivary tissue [3].

### Statistical Analysis

All statistical analyses were performed with SPSS version 18.0 (SPSS, Chicago, IL). The interobserver reproducibility was evaluated by calculating the intraclass correlation coefficient (ICC). It is between 0 and 1; the closer the value is to 1, the higher the correlation of units, as well as better repeatability. Data were presented as mean ± standard deviation (SD). The normality of data distribution was verified using the Kolmogorov–Smirnov test. For normally distributed data, the differences between groups were evaluated using the Student *t* test. The Mann-Whitney U test was used when the data were not normally distributed. Gender frequency was compared with the 2-tailed Fisher exact test. Univariate and multivariate logistic regression analyses were performed to identify significant factors associated with abnormally reduced pRNFL and mGCIPL thicknesses. Variables with *P* < 0.10 in univariate analyses were included in the multivariate analyses. Correlations between the OCT parameters and clinical factors were analyzed by Pearson correlation coefficients. *P* values of < 0.05 were considered statistically significant.

## Results

Of the 156 patients who were initially enrolled, 15 patients were excluded because of poor OCT image quality, 5 patients because of presentation of glaucomatous changes, and 6 patients because of incomplete clinical data. Finally, this study included 130 eyes of 130 patients with pSS who underwent serologic tests, minor salivary gland biopsy, and OCT examination. All ICC values of the retinal thickness measurements were above 0.95 (data not shown).

Table 1 presents the clinical characteristics of the included patients. The patients were predominantly female (97.0%). The mean age at visit and at the pSS onset was 50.28 ± 11.70 years and 44.17 ± 9.75 years, respectively. The mean duration of the disease (patient reported) was 6.12 ± 6.34 years. Most patients (97.7%) were positive for ANA, 116 patients (89.2%) were positive for anti-SSA antibody, and 47 patients (36.2%) were positive for anti-SSB antibody. All patients who were positive for anti-SSB antibody were also positive for anti-SSA. LFS was <1 in 73 patients (56.2%) and ≥1 in 57 patients (43.8%). pRNFL and mGCIPL thicknesses were abnormally reduced in 22 patients (16.9%) and 33 patients (25.4%), respectively. All patients who had abnormally reduced pRNFL thickness also had abnormally reduced mGCIPL thickness.

**Table 1:** Demographics and clinical characteristics of study patients.

	Patients (N = 130)
Mean age at visit, years	50.28 ± 11.70
Mean age at onset, years	44.17 ± 9.75
Disease duration, years	6.12 ± 6.34
Male/Female	4/126
Phakia/Pseudophakia	121/9
BCVA, logMAR	0.03 ± 0.08
SE refractive error, diopter	-0.68 ± 1.84
IOP, mmHg	14.26 ± 3.30
Humphrey visual field	
MD, dB	-0.97 ± 1.89
PSD, dB	1.84 ± 1.09
VFI, %	97.31 ± 2.92
Laboratory test	
WBC, 10^3^/μl	5.60 ± 2.23
Lymphocyte, 10^3^/μl	1.82 ± 0.64
ESR, mm/hr	34.84± 23.49
CRP, mg/dL	0.28 ± 1.11
Immunoglobulin, mg/dl	
Ig G, g/L	18.99 ± 6.08
Ig M, g/L	1.50 ± 1.19
Ig A, g/L	3.30 ± 1.20
Autoimmune lab	
ANA positive, n (%)	127 (97.7)
Anti-SSA positive, n (%)	116 (89.2)
Anti-SSA level, U/mL	154.18 ± 74.12
Anti-SSB positive, n (%)	47 (36.2)
Anti-SSB level, U/mL	44.07 ± 69.10
RF positive (%)	68 (52.3)
RF, IU/mL	47.20 ± 68.14
LFS, n (%)	0.92 ± 1.19
Score <1	73 (56.2)
Score ≥ 1	57 (43.8)
OCT, n (%)	
Abnormally reduced pRNFL	22 (16.9)
Abnormally reduced mGCIPL	33 (25.4)

BCVA, best-corrected visual acuity; LogMAR, logarithm of the minimal angle of resolution; IOP, intraocular pressure; SE, spherical equivalent; MD, mean deviation; PSD, pattern standard deviation; VFI, visual field index; WBC, white blood cell; ESR, erythrocyte sedimentation rate; CRP, C-reactive protein; D, diopters; Ig, Immunoglobulin; ANA, antinuclear antibody; Anti-SSA/SSB, anti-Sjögren syndrome A and/or B antibodies; RF, rheumatoid factor; LFS, lymphocytic focus score; OCT, optical coherence tomography; pRNFL, peripapillary retinal nerve fiber layer; mGCIPL, macular ganglion cell-inner plexiform layer.

Table 2 shows a comparison of serologic and histopathologic factors between pSS patients with normal and abnormal pRNFL thickness or normal and abnormal mGCIPL thickness. Compared with patients with normal pRNFL thickness, a significantly higher proportion of anti-SSB antibody positivity (*P* = 0.006) and higher levels of anti-SSB antibody (*P* < 0.001) was observed in patients with abnormally reduced pRNFL thickness. Similarly, significantly higher levels of ESR (*P* = 0.001), higher proportion of anti-SSB antibody positivity (*P* < 0.001), and higher levels of anti-SSB antibody (*P* < 0.001) were observed in patients with abnormally reduced mGCIPL.

**Table 2:** Comparison of serologic and histopathologic values between pSS patients with normal and abnormal pRNFL or mGCIPL thicknesses.

	pRNFL			mGCIPL		
	Normal (N = 108)	Abnormal (N = 22)	*P* value	Normal (N = 97)	Abnormal (N = 33)	*P* value
Age, years	50.08 ± 10.74	51.27 ± 15.90	0.666	46.85 ± 10.45	51.58 ± 14.91	0.465
Disease duration, years	5.84 ± 5.87	7.45 ± 8.69	0.285	5.94 ± 5.85	6.64 ± 7.95	0.592
Laboratory test						
WBC, 10^3^/μl	5.81 ± 2.30	4.90 ± 1.79	0.163	5.82 ± 2.50	5.05 ± 2.24	0.766
Lymphocyte, 10^3^/μl	1.86 ± 0.65	1.62 ± 0.58	0.105	1.85 ± 0.67	1.74 ± 0.56	0.401
ESR, mm/hr	33.48 ± 22.53	41.50 ± 27.33	0.145	31.01 ± 20.98	46.09 ± 26.98	**0.001**
CRP, mg/dL	0.28 ± 1.16	0.30 ± 0.80	0.945	0.27 ± 1.22	0.31 ± 0.70	0.861
Immunoglobulin						
IgG, g/L	18.49 ± 6.01	21.45 ± 5.97	0.060	18.50 ± 4.30	20.34 ± 7.33	0.157
IgM, g/L	1.57 ± 1.28	1.16 ± 0.43	0.179	1.48 ± 1.07	1.57 ± 1.53	0.739
IgA, g/L	3.20 ± 1.14	3.76 ± 1.40	0.075	3.18 ± 1.17	3.67 ± 1.25	0.073
Autoimmune lab						
ANA positive, n (%)	106 (98.1)	21 (95.5)	0.429	95 (97.9)	32 (97.0)	0.999
Anti-SSA positive, n (%)	95 (88.0)	21 (95.5)	0.462	85 (87.6)	31 (93.9)	0.516
Anti-SSA level, U/mL	151.93 ± 76.27	165.25 ± 62.87	0.444	147.89 ± 76.92	172.67 ± 62.63	0.097
Anti-SSB positive, n (%)	33 (30.6)	14 (63.6)	**0.006**	26 (26.8)	21 (63.6)	**<0.001**
Anti-SSB level, U/mL	33.66 ± 60.51	95.20 ± 85.90	**<0.001**	28.58 ± 55.14	89.62 ± 85.20	**<0.001**
RF positive, n (%)	57 (52.8)	11 (50.0)	0.820	52 (53.6)	16 (48.5)	0.688
RF, IU/mL	43.94 ± 70.09	59.59 ± 60.31	0.376	47.97 ± 73.03	45.41 ± 56.08	0.872
LFS	0.96 ± 1.21	0.73 ± 1.12	0.400	0.90 ± 1.18	1.00 ± 1.25	0.669

pRNFL, peripapillary retinal nerve fiber layer; mGCIPL, macular ganglion cell-inner plexiform layer; WBC, white blood cell; ESR, erythrocyte sedimentation rate; CRP, C-reactive protein; Ig, Immunoglobulin; ANA, antinuclear antibody; Anti-SSA/SSB, anti-Sjögren syndrome A and/or B antibodies; RF, rheumatoid factor; LFS, lymphocytic focus score.

Statistically significant values are in bold.

Among various clinical factors, anti-SSB antibody positivity was associated with abnormally reduced RNFL thickness in univariate (odd ratio (OR): 3.977, 95% confidence interval (CI) = 1.522–10.391, *P* = 0.005) and multivariate (OR: 3.155, 95% CI = 1.009–9.870, *P* = 0.048) logistic regression analyses (Table 3).

**Table 3:** Factors associated with abnormally reduced pRNFL thickness.

Parameter	Univariate OR (95% CI)	*P* value	Multivariate OR (95% CI)	*P* value
Age	1.009 (0.970–1.050)	0.663		
Onset age	0.983 (0.893–1.082)	0.731		
LFS	0.836 (0.552–1.267)	0.399		
WBC	0.999 (0.998–1.000)	0.124		
Lymphocyte	0.999 (0.999–1.000)	0.108		
ESR	1.013 (0.995–1.032)	0.149		
CRP	1.014 (0.683–1.507)	0.944		
IgG	1.001 (1.000–1.002)	0.057	1.001 (1.000–1.002)	0.203
IgM	0.993 (0.984–1.002)	0.125		
IgA	1.004 (1.000–1.007)	0.083	1.003 (0.998–1.007)	0.228
ANA positivity	0.396 (0.034–4.572)	0.458		
Anti–SSA positivity	2.874 (0.356–23.190)	0.322		
Anti–SSB positivity	3.977 (1.522–10.391)	**0.005**	3.155 (1.009–9.870)	**0.048**
RF positivity	1.535 (0.607–3.881)	0.365		

pRNFL, peripapillary retinal nerve fiber layer; OR, odd ratio; CI, confidence interval; LFS, lymphocytic focus score; WBC, white blood cell; Ig, Immunoglobulin; RF, rheumatoid factor; ANA, antinuclear antibody; Anti–SSA/SSB, anti–Sjögren’s syndrome A and/or B antibodies; ESR, erythrocyte sedimentation rate; CRP, C–reactive protein.

Statistically significant values are in bold.

Anti-SSB antibody positivity (OR: 4.779, 95% CI = 2.064–11.065, *P* < 0.001) and ESR level (OR: 1.026, 95% CI = 1.009–1.044, *P* = 0.003) were significantly associated with abnormally reduced mGCIPL thickness in univariate logistic regression analysis. In multivariate logistic regression analysis, anti-SSB antibody positivity (OR: 4.175, 95% CI = 1.557–11.195, *P* = 0.005), and ESR levels (OR: 1.026, 95% CI = 1.002–1.051, *P* = 0.031) were associated with abnormally reduced mGCIPL thickness (Table 4).

**Table 4:** Factors associated with abnormally reduced mGCIPL thickness.

Parameter	Univariate OR (95% CI)	*P* value	Multivariate OR (95% CI)	*P* value
Age	1.013 (0.979–1.048)	0.462		
Onset age	1.026 (0.947–1.112)	0.528		
LFS	1.075 (0.774–1.491)	0.667		
WBC	1.000 (0.999–1.000)	0.367		
Lymphocyte	1.000 (0.999–1.000)	0.398		
ESR	1.026 (1.009–1.044)	**0.003**	1.026 (1.002–1.051)	**0.031**
CRP	1.030 (0.738–1.440)	0.860		
IgG	1.001 (1.000–1.001)	0.160		
IgM	1.001 (0.997–1.004)	0.737		
IgA	1.003 (1.000–1.007)	0.080	1.001 (0.996–1.005)	0.764
ANA positivity	0.674 (0.059–7.680)	0.750		
Anti–SSA positivity	2.188 (0.463–10.335)	0.323		
Anti–SSB positivity	4.779 (2.064–11.065)	**<0.001**	4.175 (1.557–11.195)	**0.005**
RF positivity	1.151 (0.511–2.593)	0.734		

mGCIPL, macular ganglion cell–inner plexiform layer pRNFL, peripapillary retinal nerve fiber layer; OR, odd ratio; CI, confidence interval; LFS, lymphocytic focus score; WBC, white blood cell; Ig, Immunoglobulin; RF, rheumatoid factor; ANA, antinuclear antibody; Anti–SSA/SSB, anti–Sjögren’s syndrome A and/or B antibodies; ESR, erythrocyte sedimentation rate; CRP, C–reactive protein.

Statistically significant values are in bold.

**Fig 2:**
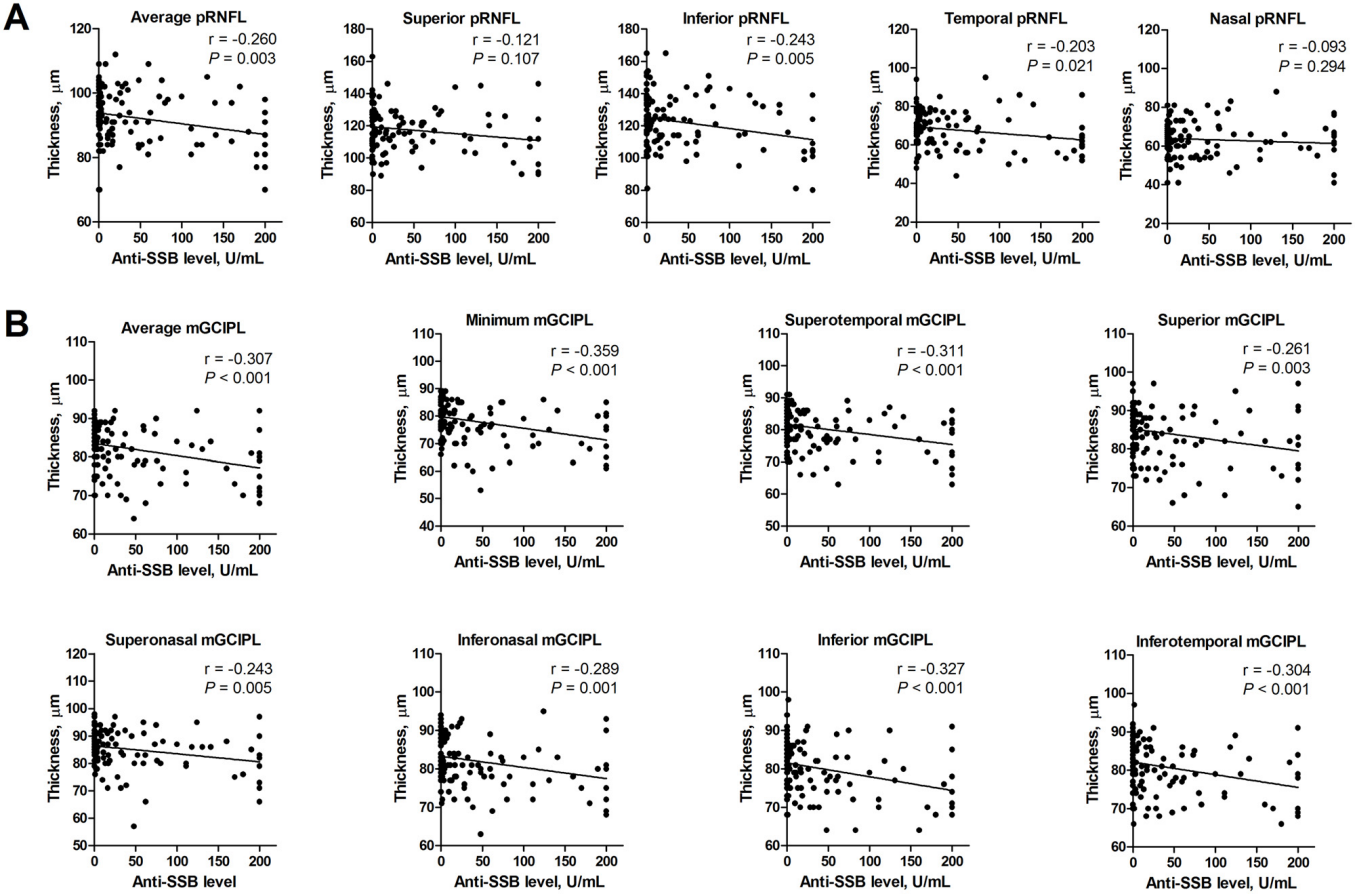
Scatterplot and linear regression line showing the relationship between OCT measurements and anti-SSB antibodies in patients with primary Sjögren's syndrome. (A) Average, inferior, and temporal pRNFL thicknesses were significantly correlated with the levels of anti-SSB (*P* < 0.05). (B) All the parameters of mGCIPL thicknesses were significantly correlated with the anti-SSB levels (*P* < 0.01).

Fig 2 shows scatterplots of the correlation between the pRNFL or mGCIPL thicknesses and the level of anti-SSB antibody. Anti-SSB antibody levels showed a significant negative correlation with the average (r = −0.260; *P* = 0.003), inferior (r = −0.243; *P* = 0.005), and temporal (r = −0.203; *P* = 0.021) pRNFL thicknesses, and with all parameters of the mGCIPL thickness (all *P* < 0.01); the strongest correlation was observed with the minimum mGCIPL thickness (r = −0.359; *P* < 0.001). ESR level showed a weak negative correlation with the average pRNFL thickness (r = −0.180; *P* = 0.041) and inferior mGCIPL thickness (r = −0.177; *P* = 0.045) (S1 Table). However, there was no statistically significant relationship between functional outcome (BCVA and VF indices) and OCT parameters (S2 and S3 Tables).

**Fig 3:**
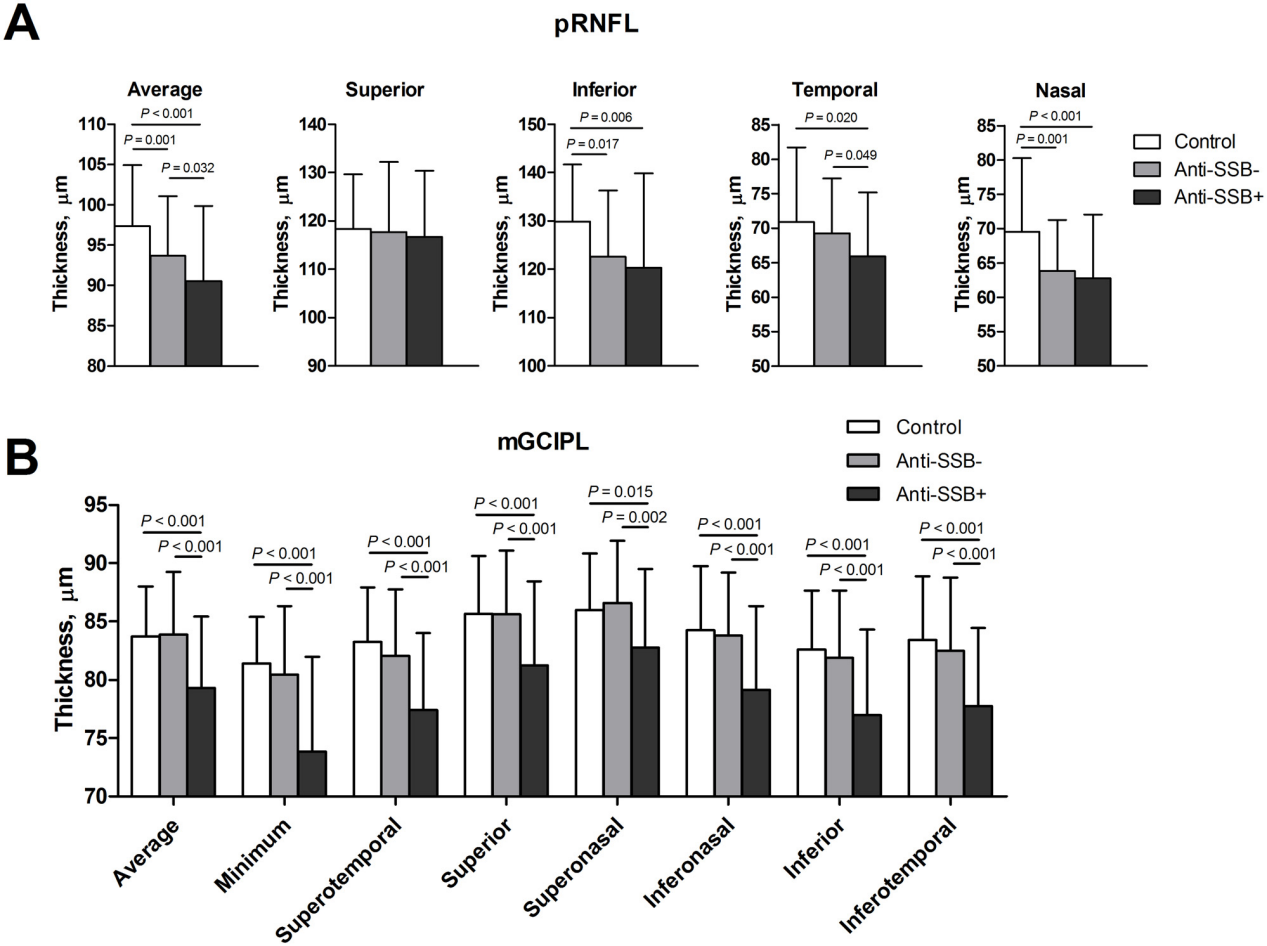
Comparison of pRNFL and mGCIPL thicknesses according to anti-SSB positivity in patients with primary Sjögren's syndrome. (A) Comparison of pRNFL thicknesses between groups. (B) Comparison of mGCIPL thicknesses between groups.

Fig 3 shows a comparison of mean retinal thicknesses according to anti-SSB positivity. Although there were no statistically significant differences in age, disease duration, sex, IOP, SE refractive error, and BCVA between controls and subgroups (S4 Table), patients who were positive for anti-SSB antibodies showed significantly thinner average (vs. control, *P* < 0.001; vs. anti-SSB negative, *P* = 0.032), inferior (vs. control, *P* = 0.006), temporal (vs. control, *P* = 0.020; vs. anti-SSB negative, *P* = 0.049), and nasal (vs. control, *P* < 0.001) pRNFL thickness and all parameters of the mGCIPL thickness (vs. control, all *P* < 0.02; vs. anti-SSB negative, all *P* < 0.01) in comparison with control and patients who were negative for anti-SSB antibody.

## Discussion

Measurement of retinal thickness provided by OCT has been a popular auxiliary method for studying retinal pathology in neurodegenerative diseases and also in vascular diseases of the CNS [25–27]. In recent studies, OCT has been widely used as a tool for studying the disease pathology and differentiation: detection of subtle retinal abnormalities caused by vascular changes in idiopathic moyamoya angiopathy, differentiation of Susac syndrome from multiple sclerosis, and detection of localized RNFL defect caused by ischemic injury of nerve fiber layer in Behcet’s disease [25,26,28]. Moreover, segmentation of retinal layers had provided a deeper understanding of the disease pathology. Injury of the RGC or the axons traveling within the pRNFL caused by inflammation or aberrant autoimmunity may result in the thinning of the mGCIPL and pRNFL layer which is reflected in the OCT [14,29–31]. Therefore, incorporated into the paraclinical data, OCT findings might provide additional insights into the pathophysiology of retinal neurodegeneration in pSS [14,27].

In this study, serum anti-SSB positivity was associated with abnormally reduced pRNFL thickness. In addition to anti-SSB antibody positivity, elevated ESR level was associated with abnormally reduced mGCIPL thickness in patients with pSS. We also observed an apparent negative correlation between the levels of anti-SSB antibodies and inner retinal thickness (pRNFL and mGCIPL). Patients that were anti-SSB-positive showed significant thinning of pRNFL and mGCIPL when compared to patients who were anti-SSB-negative. These findings are in line with our previous study, which showed significant thinning of pRNFL and mGCIPL in patients with pSS [14].

Anti-SSB antibodies are more common in patients with systemic organ involvement than in patients with no such involvement [6]. In general, higher titers of anti-SSA and anti-SSB antibodies indicate a more active disease with more systemic manifestations in patients with pSS [32–34]. ESR is also significantly associated with disease activity of pSS and indicates the presence of active inflammation [35,36]. Therefore, our results suggest that increased anti-SSB antibody positivity and levels indicate increased immune dysregulation, which may lead to greater neurodegenerative changes in the inner retina in patients with pSS. In addition, this neurodegenerative change could be more pronounced in a patient subgroup with increased ESR levels, which indicates active inflammatory status.

The serological features of the disease and the importance of anti-SSB have been stressed as markers of active inflammatory infiltration in the salivary gland, earlier disease onset, and larger number of extraglandular manifestations [7]. On the contrary, anti-SSA is not entirely specific for pSS which might limit its utility in reflecting pSS disease-specific immunoactivity, since it has been shown to be associated with various subsets of autoimmune disorders [5,37]. We hypothesized that, similar to its role in the infiltration of exocrine glands, higher serum levels of anti-SSB rather than anti-SSA would closely relate to the development of dysregulated immunity which might then be reflected in the inner retinal structure [38]. The presence of anti-SSB indicates a subset of patients with pSS that shows T cell dysfunction in the peripheral blood [39]. Peripheral blood T cell dysfunction and trafficking of immune cells is known to interrelate with higher intensity of immune response at the site of inflammation including exocrine glands and other organs [39–41]. Therefore, we suggest that serums levels of anti-SSB rather than anti-SSA could represent a reliable marker that reflects the fluctuation of immune response which might affect the microenvironment of the inner retina including RGCs.

Regarding the role of aberrant autoimmunity in RGC injury, a possible pathomechanism in patients with pSS can be suggested on the basis of the results of previous studies that used the neonatal heart block model: (1) maternal anti-SSA/SSB autoantibodies cross the placenta and form IgG–apoptotic cell complexes and (2) apoptotic cardiocytes are opsonized by anti-SSA/SSB autoantibodies, which impairs clearance by normal cardiocytes [42–44]. Likewise, during normal tissue turnover, binding of these autoantibodies to apoptotic RGCs could impair clearance of these cells, which in turn may elicit proinflammatory responses including macrophage infiltration and increased production of interleukin-1, tumor necrosis factor α, endothelin-1, and nitric oxide, ultimately leading to RGC damage [45–47]. This was further supported by a study by Gramilch et al.[48], which revealed IgG autoantibody accumulation and increased levels of proinflammatory cytokines in human glaucomatous retina. Therefore, dysregulated apoptosis and proinflammatory conditions, indicated by increased serum autoantibody levels, may promote a neurodegeneration in patients with pSS.

Generally, patients with higher LFS are more likely to present systemic extraglandular manifestations [9,49,50]. These patients are expected to show increased serum cytokine levels and autoantibodies, including anti-SSA/SSB; LFS is known to be significantly correlated with the severity of inflammation and the production of anti-SSA/SSB antibodies [9,51]. Our study also confirmed a positive correlation between LFS and the levels of anti-SSA/SSB antibodies (data not shown). Therefore, we expected that LFS would be significantly correlated with neurodegeneration in retina. However, LFS was not associated with abnormally reduced pRNFL or mGCIPL thickness and showed no significant correlation with pRNFL or mGCIPL thickness. Although we have no definite explanation for this finding, it might be attributed to the differing methodologies used to evaluate retinal thickness or lymphocytic infiltration of salivary tissues. Further studies involving larger numbers of patients and using advanced histological methods are warranted to clarify this issue.

Previous studies have indicated that the significant relationship between structure and functional outcome is limited only in cases of advanced glaucoma [52,53]. Although our study includes patients with abnormally reduced pRNFL and mGCIPL thicknesses, only mild structural changes were observed due to the exclusion of patients with conditions such as glaucoma (ie, shows abnormal results in the visual field). Consequently, we could not demonstrate a significant relationship between structure (OCT parameters) and functional outcomes (BCVA, visual field indices). These observations are relevant in the context of early-stage glaucoma, especially in the preperimetric glaucoma stage where no significant structure-function associations are observed [52,53]. Nonetheless, our study provides a noteworthy contribution in revealing that pSS patients with no direct involvement of the CNS could show retinal structural changes resulting from an increase in autoimmunity; this abnormally reduced pRNFL and mGCIPL thicknesses might reflect the vulnerability of the cytotoxic drug therapy-induced RGC injury. Our findings have practical relevance in the management of patients with pSS; further enforcement studies may help validate whether these structural changes would be useful in predicting CNS involvement or the vulnerability of RGC loss caused by a cytotoxic therapy

The strengths of the present study are putting the parameters for predicting inner retinal changes in the context of routine laboratory examinations of patients with pSS. However, our study also has several limitations. First, as is usually found in retrospective studies, we cannot exclude a selection bias. Second, due to the high prevalence of females among patients with pSS, mainly females were included. Third, we did not measure the axial lengths of our patients’ eyes. However, most of our patients were emmetropic and also, there was no statistically significant difference in SE between patients with positive anti-SSB autoantibody and negative anti-SSB autoantibody; therefore, the axial length might not considerably affect the measurements of pRNFL and mGCIPL thicknesses. Fourth, we could not evaluate genetic information, such as leukocyte antigen type, or environmental factors affecting the subjects. Genetic and environmental factors play a crucial role in autoimmune diseases; therefore, our results may not be representative of other racial populations. Lastly, as our study was a retrospective cross-sectional design, we could not provide any longitudinal data associated with the progression of the retinal changes.

In conclusion, anti-SSB autoantibodies might be a useful marker to predict abnormally reduced pRNFL and mGCIPL thickness in patients with pSS. Patients with higher levels of anti-SSB antibodies could be prone to greater neurodegeneration in the inner retina. Given this association, the need for close monitoring of inner retinal changes in this population is stressed, especially in those undergoing high-dose retinal toxic medication. Taken together, the current findings may further assist clinicians in identification of pSS patients who are at risk of developing neurodegeneration in the inner retina and should be closely monitored. Further longitudinal studies with larger patient populations will be needed to clarify the risk of progression of retinal morphological changes in patients with pSS.

## Supporting Information

S1 Table Correlation Coefficients Between OCT Measurements and anti-SSB antibody and ESR level in Patients with Primary Sjögren's Syndrome. (DOCX)

S2 Table Correlation Coefficients Between OCT Measurements and BCVA in Patients with Primary Sjögren's Syndrome. (DOCX)

S3 Table Correlation Coefficients Between OCT Measurements and Visual field indices in Patients with Primary Sjögren's Syndrome. (DOCX)

S4 Table Comparison of pRNFL and mGCIPL Thicknesses according to anti-SSB Positivity in Patients with Primary Sjögren's Syndrome. (DOCX)
