# Supplementary material for: Analysis of Clinical Factors Associated with Retinal Morphological Changes in Patients with Primary Sjögren's Syndrome
Source: PLoS One. 2016 Jun 21;11(6):e0157995. doi: 10.1371/journal.pone.0157995 (PMC4915668; doi:10.1371/journal.pone.0157995)
Supplement: S1 Table — (DOCX) [file pone.0157995.s001.docx]

**S1 Table.** Correlation Coefficients Between OCT Measurements and anti-SSB antibody and ESR level in Patients with Primary Sjögren's Syndrome

|  | **Anti-SSB level** | | **ESR level** | |
| --- | --- | --- | --- | --- |
|  | ***r*** | ***P*** | ***r*** | ***P*** |
| pRNFL thickness | |  |  |  |
| Average | -0.260 | **0.003** | -0.180 | **0.041** |
| Superior | -0.121 | 0.107 | -0.140 | 0.111 |
| Inferior | -0.243 | **0.005** | -0.047 | 0.598 |
| Temporal | -0.203 | **0.021** | -0.134 | 0.127 |
| Nasal | -0.093 | 0.294 | -0.089 | 0.315 |
| mGCIPL thickness | |  |  |  |
| Average | -0.307 | **<0.001** | -0.110 | 0.214 |
| Minimum | -0.359 | **<0.001** | -0.099 | 0.264 |
| Superotemporal | -0.311 | **<0.001** | -0.065 | 0.460 |
| Superior | -0.261 | **0.003** | -0.090 | 0.310 |
| Superonasal | -0.243 | **0.005** | -0.085 | 0.335 |
| Inferonasal | -0.289 | **0.001** | -0.074 | 0.404 |
| Inferior | -0.327 | **<0.001** | -0.177 | **0.045** |
| Inferotemporal | -0.304 | **<0.001** | -0.102 | 0.247 |

OCT, optical coherence tomography; Anti-SSB, anti-Sjögren’s syndrome B antibodies; ESR, erythrocyte sedimentation rate; pRNFL, peripapillary retinal nerve fiber layer; mGCIPL, macular ganglion cell-inner plexiform layer

Statistically significant values are in bold.
