# Supplementary material for: Analysis of Clinical Factors Associated with Retinal Morphological Changes in Patients with Primary Sjögren's Syndrome
Source: PLoS One. 2016 Jun 21;11(6):e0157995. doi: 10.1371/journal.pone.0157995 (PMC4915668; doi:10.1371/journal.pone.0157995)
Supplement: S2 Table — (DOCX) [file pone.0157995.s002.docx]

**S2 table.** Correlation Coefficients Between OCT Measurements and BCVA in Patients with Primary Sjögren's Syndrome

|  | **BCVA** | | | |
| --- | --- | --- | --- | --- |
|  | **Anti-SSB-** | | **Anti-SSB+** | |
|  | ***r*** | ***P*** | ***r*** | ***P*** |
| pRNFL thickness | |  |  |  |
| Average | -0.084 | 0.452 | 0.031 | 0.836 |
| Superior | -0.009 | 0.935 | 0.032 | 0.833 |
| Inferior | 0.008 | 0.942 | 0.127 | 0.396 |
| Temporal | 0.057 | 0.606 | -0.043 | 0.775 |
| Nasal | -0.036 | 0.747 | -0.038 | 0.800 |
| mGCIPL thickness | |  |  |  |
| Average | 0.135 | 0.224 | 0.191 | 0.199 |
| Minimum | 0.138 | 0.212 | 0.104 | 0.485 |
| Superotemporal | 0.167 | 0.132 | 0.173 | 0.245 |
| Superior | 0.085 | 0.447 | 0.169 | 0.265 |
| Superonasal | 0.093 | 0.404 | 0.184 | 0.215 |
| Inferonasal | 0.158 | 0.153 | 0.237 | 0.108 |
| Inferior | 0.103 | 0.356 | 0.085 | 0.572 |
| Inferotemporal | 0.170 | 0.124 | 0.161 | 0.280 |

OCT, optical coherence tomography; BCVA, best-corrected visual acuity; Anti-SSB, anti-Sjögren’s syndrome B antibodies; pRNFL, peripapillary retinal nerve fiber layer; mGCIPL, macular ganglion cell-inner plexiform layer
