# Supplementary material for: Analysis of Clinical Factors Associated with Retinal Morphological Changes in Patients with Primary Sjögren's Syndrome
Source: PLoS One. 2016 Jun 21;11(6):e0157995. doi: 10.1371/journal.pone.0157995 (PMC4915668; doi:10.1371/journal.pone.0157995)
Supplement: S3 Table — (DOCX) [file pone.0157995.s003.docx]

**S3 table.** Correlation Coefficients Between OCT Measurements and Visual field indices in Patients with Primary Sjögren's Syndrome

|  | **MD** | | | | | **PSD** | | | | | **VFI** | | | |
| --- | --- | --- | --- | --- | --- | --- | --- | --- | --- | --- | --- | --- | --- | --- |
|  | **Anti-SSB-** | | **Anti-SSB+** | | **Anti-SSB-** | | | **Anti-SSB+** | | **Anti-SSB-** | | | **Anti-SSB+** | |
|  | ***r*** | ***P*** | ***r*** | ***P*** | ***r*** | | ***P*** | ***r*** | ***P*** | ***r*** | | ***P*** | ***r*** | ***P*** |
| pRNFL thickness | | | | | | | | | | | | | | |
| Average | 0.002 | 0.985 | 0.027 | 0.876 | -0.080 | | 0.510 | -0.003 | 0.987 | -0.005 | | 0.963 | 0.014 | 0.933 |
| Superior | -0.077 | 0.525 | -0.034 | 0.844 | 0.039 | | 0.751 | -0.057 | 0.743 | 0.020 | | 0.862 | -0.100 | 0.543 |
| Inferior | 0.044 | 0.716 | 0.058 | 0.735 | -0.008 | | 0.946 | 0.064 | 0.709 | 0.071 | | 0.538 | 0.108 | 0.511 |
| Temporal | 0.010 | 0.934 | 0.118 | 0.494 | -0.131 | | 0.281 | -0.107 | 0.536 | -0.100 | | 0.383 | 0.239 | 0.142 |
| Nasal | 0.003 | 0.983 | -0.111 | 0.519 | 0.040 | | 0.745 | 0.001 | 0.998 | -0.026 | | 0.818 | 0.034 | 0.835 |
| mGCIPL thickness | | | | | | | | | | | | | | |
| Average | 0.011 | 0.927 | -0.143 | 0.406 | -0.022 | | 0.858 | 0.052 | 0.761 | 0.096 | | 0.401 | 0.090 | 0.588 |
| Minimum | 0.088 | 0.469 | -0.195 | 0.255 | 0.029 | | 0.813 | 0.120 | 0.487 | 0.099 | | 0.388 | 0.019 | 0.906 |
| Superotemporal | 0.084 | 0.841 | -0.218 | 0.202 | -0.100 | | 0.412 | 0.089 | 0.607 | 0.111 | | 0.332 | 0.005 | 0.978 |
| Superior | -0.005 | 0.967 | -0.132 | 0.444 | -0.053 | | 0.661 | 0.068 | 0.693 | 0.068 | | 0.552 | 0.067 | 0.685 |
| Superonasal | -0.013 | 0.914 | -0.067 | 0.697 | 0.056 | | 0.645 | 0.044 | 0.797 | 0.020 | | 0.859 | 0.183 | 0.266 |
| Inferonasal | 0.043 | 0.726 | -0.078 | 0.651 | -0.013 | | 0.915 | -0.008 | 0.962 | 0.078 | | 0.495 | 0.141 | 0.393 |
| Inferior | 0.018 | 0.885 | -0.122 | 0.477 | 0.029 | | 0.809 | 0.057 | 0.740 | 0.089 | | 0.438 | 0.006 | 0.969 |
| Inferotemporal | 0.024 | 0.841 | -0.114 | 0.508 | -0.004 | | 0.977 | 0.046 | 0.789 | 0.122 | | 0.288 | -0.054 | 0.745 |

OCT, optical coherence tomography; MD, mean deviation; PSD, pattern standard deviation; VFI, visual field index; Anti-SSB, anti-Sjögren’s syndrome B antibodies; pRNFL, peripapillary retinal nerve fiber layer; mGCIPL, macular ganglion cell-inner plexiform layer
