# Supplementary material for: Analysis of Clinical Factors Associated with Retinal Morphological Changes in Patients with Primary Sjögren's Syndrome
Source: PLoS One. 2016 Jun 21;11(6):e0157995. doi: 10.1371/journal.pone.0157995 (PMC4915668; doi:10.1371/journal.pone.0157995)
Supplement: S4 Table — (DOCX) [file pone.0157995.s004.docx]

**S4 table.** Comparison of pRNFL and mGCIPL Thicknesses according to anti-SSB Positivity in Patients with Primary Sjögren's Syndrome

| **Parameters** | **Control** | | **Anti-SSB-** | **Anti-SSB+** | | | ***P1*** | | ***P2*** | | ***P3*** |
| --- | --- | --- | --- | --- | --- | --- | --- | --- | --- | --- | --- |
|  | **Mean ± SD, (N = 73)** | | **Mean ± SD, (N = 83)** | **Mean ± SD, (N = 47)** | | |  |  |  |  |  |
| Age (years) | 52.83 ± 11.26 | | 50.41 ± 10.26 | 50.06 ± 14.01 | | | 0.267 | | 0.295 | | 0.985 |
| Disease duration (years) | N/A | | 6.57 ± 6.28 | 5.32 ± 6.67 | | | N/A | | N/A | | 0.289* |
| Sex (male/female) | 3/70 | | 2/81 | 2/45 | | | 0.665 | | 0.969 | | 0.620 |
| IOP (mmHg) | 13.90 ± 2.36 | | 14.10 ± 1.37 | 14.45 ± 1.31 | | | 0.781 | | 0.130 | | 0.781 |
| SE (D) | -0.75 ± 1.72 | | -0.75 ± 1.78 | -0.55 ± 1.94 | | | 0.999 | | 0.809 | | 0.812 |
| BCVA (LogMAR) | 0.04 ± 0.09 | | 0.02 ± 0.06 | 0.04 ± 0.10 | | | 0.213 | | 0.143 | | 0.631 |
| pRNFL thickness (μm) | |  | | | |  | |  | |  |  |
| Average | 97.38 ± 7.57 | | 93.67 ± 7.41 | 90.55 ± 9.30 | | | **0.001** | | **<0.001** | | **0.032** |
| Superior | 118.34 ± 11.29 | | 117.72 ± 14.46 | 116.68 ± 13.74 | | | 0.904 | | 0.916 | | 0.989 |
| Inferior | 129.90 ± 11.79 | | 122.59 ± 13.70 | 120.28 ± 19.57 | | | **0.017** | | **0.006** | | 0.962 |
| Temporal | 70.90 ± 10.81 | | 69.29 ± 7.94 | 65.91 ± 9.28 | | | 0.565 | | **0.020** | | **0.049** |
| Nasal | 69.55 ± 10.75 | | 63.84 ± 7.40 | 62.76 ± 9.29 | | | **0.001** | | **<0.001** | | 0.815 |
| mGCIPL thickness (μm) | |  | | | |  | |  | |  |  |
| Average | 83.71 ± 4.31 | | 83.88 ± 5.39 | 79.32 ± 6.12 | | | 0.998 | | **<0.001** | | **<0.001** |
| Minimum | 81.40 ± 4.00 | | 80.45 ± 5.89 | 73.87 ± 8.10 | | | 0.577 | | **<0.001** | | **<0.001** |
| Superotemporal | 83.27 ± 4.66 | | 82.05 ± 5.68 | 77.40 ± 6.61 | | | 0.359 | | **<0.001** | | **<0.001** |
| Superior | 85.68 ± 4.94 | | 85.63 ± 5.46 | 81.23 ± 7.22 | | | 0.998 | | **<0.001** | | **<0.001** |
| Superonasal | 85.99 ± 4.84 | | 86.60 ± 5.33 | 82.77 ± 6.75 | | | 0.807 | | **0.015** | | **0.002** |
| Inferonasal | 84.26 ± 5.48 | | 83.79 ± 5.40 | 79.15 ± 7.17 | | | 0.875 | | **<0.001** | | **<0.001** |
| Inferior | 82.59 ± 5.05 | | 81.88 ± 5.76 | 76.96 ± 7.35 | | | 0.737 | | **<0.001** | | **<0.001** |
| Inferotemporal | 83.42 ± 5.47 | | 82.48 ± 6.31 | 77.79 ± 6.65 | | | 0.601 | | **<0.001** | | **<0.001** |
| Signal strength of OCT scans | |  | | |  |  | |  | |  |  |
| Macular scan | 8.27 ± 0.56 | | 8.20 ± 0.65 | 8.10 ± 0.76 | | | 0.888 | | 0.143 | | 0.343 |
| Optic disc scan | 7.91 ± 0.64 | | 7.75 ± 0.60 | 7.79 ± 0.75 | | | 0.775 | | 0.818 | | 0.928 |

pRNFL, peripapillary retinal nerve fiber layer; mGCIPL, macular ganglion cell-inner plexiform layer; SD, standard deviation; Anti-SSB, anti-Sjögren syndrome B antibodies; IOP, intraocular pressure; SE, spherical equivalent; D, diopters; BCVA, best-corrected visual acuity; LogMAR, logarithm of the minimal angle of resolution; OCT, optical coherence tomography

*P1,* Control vs Anti-SSB-; *P2,* Control vs Anti-SSB+; *P3,* Anti-SSB- vs Anti-SSB+

One-way ANOVA with post hoc analysis with Tukey test for continuous variables.

Two-tailed Fisher exact test for categorical variable.

* Student *t* test was applied

Statistically significant values are in bold.
